# 

**Published:** 1900-03-24

**Authors:** 


### NOTICE TO CORRESPONDENTS.

All MS., Letters, Books for Review, and other matters intended for the Editor should be addressed The Editor, "The Hospital," The Hospital Building, 28 & 29, Southampton Street, Strand, W.C.

The Editor cannot undertake to return rejected MS., even when accompanied by stamped directed envelope.

**Notice to Advertisers and Subscribers.**

All Advertisements, Orders for Copies of The Hospital, and Business Communications should be addressed to **THE MANAGER (Not to the Editor)**, The Hospital, The Hospital Building, 28 & 29, Southampton Street, Strand, London, W.C.

The Cost of Subscription, post free, is as follows:—Per week, 2½d.; per quarter, 2s. 8d.; per half-year, 5s. 3d.; per annum, 10s. 6d. Foreign:—Per annum, 15s. 2d., payable, in all cases, in advance.

**NOTICE.—Vol. XXVI. of "The Hospital" (April, 1899, to September, 1899), in handsome cloth binding, is now on sale at the Office of this Journal, price 7s. 6d. Cases for binding the Numbers contained in this Volume 1s. 6d. Index to Vol. XXVI., 2½d., post free.**

**The Monthly Part for April will be ready on 2nd April. Price 6d., by post 8d.**

### NOTES ON BOOKS.

The "Municipal Year-Book for 1900," edited by Robert Donald, editor of "The Municipal Journal" (Edward Lloyd, Limited; price 2s. 6d.), is one of those useful reference books which it is almost impossible for any man, who is a citizen in the real sense of the word, to do without. The municipal work of the United Kingdom is described, much information being given in a pithy form regarding many towns. In the Irish section appears a description of the working of the new Local Government Act, which came into operation at the beginning of last year. Sections are devoted to the supply of water, gas, electricity, the management of tramways, artisans' dwellings, and municipal lodging-houses. The work involved in the editing of such a book must be of a very painstaking and laborious character, and we congratulate Mr. Donald on the successful result of his task.

Among the booklets of the hour, good at the moment but meaningless if separated from it, is "Thankful Tommy," his reply to the "Absent-Minded Beggar," a poem by R. Adams Foster, illustrated by A. R. Forrest. We confess, however, that we think more highly of the good fellow who by some strange freak has come to be called "Tommy" than we think anyone would be led to do by much of the art and literature which is nowadays poured out on his behalf.

### BOOKS RECEIVED.

Methuen and Co.
"A Handbook of Nursing." By M. N. Oxford.

Bailliere, Tindall, and Cox.
"Aids to Practical Pharmacy." By A. Campbell Stark.
"Contagious Ophthalmia, Acute and Chronic." By Sydney Stephenson.

Swan Sonnenschein and Co.
"A Primer of the Physiological Action of Alcohol." By Edwin J. Norris, M.R.C S., L.R.C.P., with an introduction by Dr. Clifford.

Adam and Charles Black.
"Who's Who at the War."

James Willing, Jun.
"Willing's Press Guide, 1900."

Adlard and Son.
"The Evolution of Asylum Architecture." By R. H. Steen, M.D. Lond.

"City Press" Office.

"The C.I.V. and the War."

J. W. Arrowsmith.
"Nordrach at Home." By Jos. J. S. Lucas, B.A., M.R.C.S., L.R.C.P.

James Bowden.
"Brain and Body: The Nervous System in Social Life." By Dr. Andrew Wilson.

Henry J. Glaisher.
"Consumption: Its Nature and Treatment." By William H. Spencer, M.A., M.D., M.R.C.P.

Society for Promoting Christian Knowledge.
"Prayers for the Use of Ward Sisters and Nurses in Hospitals and Infirmaries."

Unwin Brothers.
"Our Patent Laws." By James Keith, C.E.

Simpkin, Marshall, Hamilton, Kent, and Co.
"A Host of Thorns." By Helen Costerton Wilkinson.

The Scientific Press.
"The Art of Feeding the Invalid." By a Medical Practitioner and a Lady Professor of Cookery.

John Murray.
"Handbook of Physiology." By W. D. Halliburton, M.D., F.R.S. (Sixteenth Edition of Kirke's "Handbook of Physiology.)

**Periodicals and Pamphlets.**—Sanitary Record, Scalpel, Medical Press, Literary Digest, Leeds Hospital Magazine, Trained Nurse Review, St. Thomas's Hospital Gazette, Medical Record, Sanitary Record, Literary Digest, What Is Right, Review of the Week, Cassell's Magazine, Leisure Hour, Sunday at Home, and Boy's Own Paper.

Demy 8vo., with two Plates, cloth gilt, 12s. 6d. net.

MEDICAL HISTORY FROM THE EARLIEST TIMES.

By E. T. WITHINGTON, M.A., M.B.Oxon.

"One of the best attempts that has yet been made in the English language to present the reader with a concise epitome of the history of medicine, and as such we very cordially commend it."—*Glasgow Medical Journal.*

London: THE SCIENTIFIC PRESS, LTD., 28 & 29, SOUTHAMPTON STREET, STRAND, W.C.

NOW READY,

A REPRINT OF

A NURSING HANDBOOK

TOGETHER WITH S. MAW, SON, AND THOMPSON'S

COMPLETE CATALOGUE OF NURSING REQUISITES.

The above work is now republished at a cost of 1s. per copy. Messrs. S. M., S., and T. have been compelled to make this charge on account of the extreme popularity of the work and the great expense incurred in its production.

*Post Free on receipt of 1s.*

S. MAW, SON, and THOMPSON,

7 to 12, ALDERSGATE STREET, LONDON, E.C.

